# Supplementary material for: The study of GSDMB in pathogenesis of psoriasis vulgaris
Source: PLoS One. 2023 Jan 6;18(1):e0279908. doi: 10.1371/journal.pone.0279908 (PMC9821418; doi:10.1371/journal.pone.0279908)
Supplement: S1 File — (PDF) [file pone.0279908.s001.pdf]

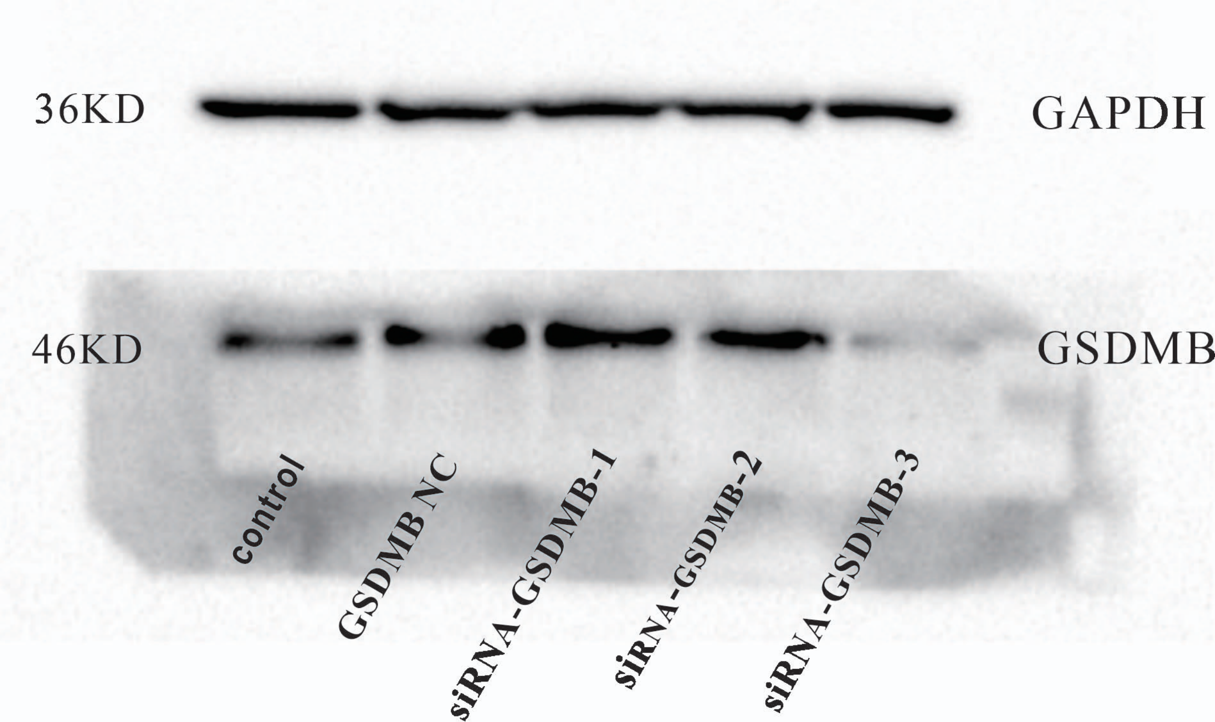
36KD
GAPDH
46KD
GSDMB
control
GSDMB NC
siRNA-GSDMB-1
siRNA-GSDMB-2
siRNA-GSDMB-3

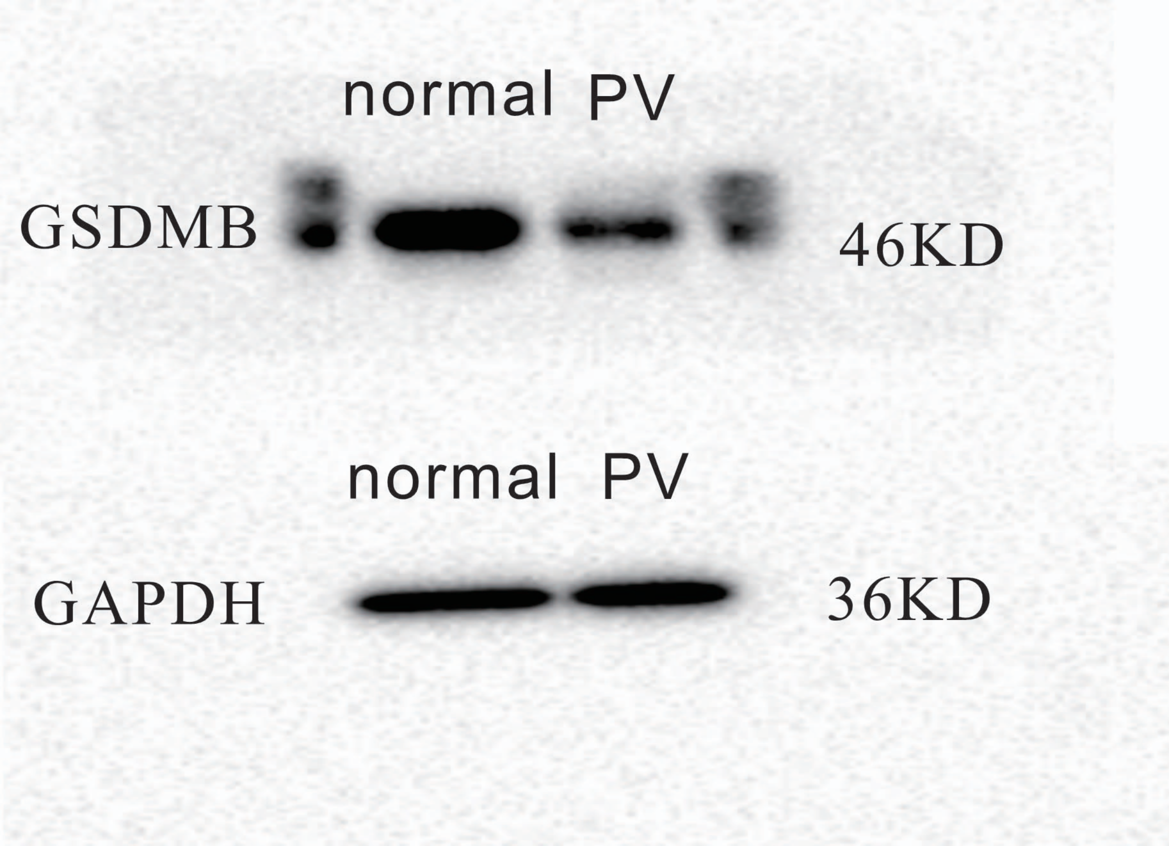
normal PV
GSDMB
46KD
normal PV
GAPDH
36KD
