# Supplementary material for: The study of GSDMB in pathogenesis of psoriasis vulgaris
Source: PLoS One. 2023 Jan 6;18(1):e0279908. doi: 10.1371/journal.pone.0279908 (PMC9821418; doi:10.1371/journal.pone.0279908)
Supplement: Attachment — Submitted filename: Comments-Plos one.docx [file pone.0279908.s002.docx]

The authors investigated the role of GSDMB in the pathogenesis of psoriasis. This brings new insights into the pathophysiology of the psoriasis and clinical investigation of the GSDMB. Here are some comments to improve the impact of the article.

Minor comment:
1) The author mentioned GSDMB’s role in cell pyrolysis process (Line 26), it will better to clary the potential effects of pyrolysis on psoriasis, since there are no reports on pyrolysis in psoriasis. Do you mean proptosis? If so, please correct it.

2) Line 102, CO^2^ should be CO_2_

3) Lines 265-266, the author said “GSDMB is not involved in the pathogenesis of psoriasis by affecting keratinocyte proliferation/apoptosis”, but the results showed GSDMB can promote HaCaT proliferation and inhibit apoptosis. Can author verify the results and conclusion?

4) References 12, 22, and 24, the author names are capital, inconsistent with other ones.

5) The figures are low-quality, please replace higher ones.
